# MEF2B is the ideal immunohistochemical marker to highlight neoplastic LP cells in nodular lymphocyte‐predominant Hodgkin lymphoma

**DOI:** 10.1002/jha2.690

**Published:** 2023-04-13

**Authors:** Alireza Torabi, Jonathan R Fromm, Kikkeri N Naresh

**Affiliations:** ^1^ Department of Laboratory Medicine and Pathology Division of Hematopathology University of Washington Seattle Washington USA; ^2^ Pathology Program Translational Science and Therapeutics Division Fred Hutchinson Cancer Research Center Seattle Washington USA; ^3^ Department of Laboratory Medicine and Pathology University of Washington Seattle Washington USA

1

Nodular lymphocyte predominant Hodgkin lymphoma (NLPHL) is a tumor composed of scattered germinal center (GC) derived large neoplastic B‐cells with multilobulated nuclei (LP cells) in a background of abundant reactive B‐ and T‐cells and histiocytes within a follicular environment [1]. Neoplastic cells express B‐cell markers (CD20, CD79a, CD19, PAX5, OCT2, BOB1, PU‐1, and J‐chain) and some GC markers such as BCL6, LMO2, HGAL, and MEF2B (myocyte enhancer‐binding factor 2B); neoplastic cells in a subset of NLPHL also express IgD. Neoplastic LP cells can be negative for B‐cell markers CD19, CD20, or CD79a. The background benign B‐cells are mostly mantle cells (expressing all B‐cell markers along with IgM and IgD). There are abundant benign CD4+ T‐cells, most of which are T‐follicular‐helper cells (TFH cells) expressing BCL6, PD1, and other TFH markers. In its characteristic presentation, the nodules show prominent follicular dendritic cell meshworks highlighted by antibodies such as CD21 or CD23 [2, 3, 4, 5].

Although LP cells in most cases of NLPHL express all B‐cell antigens, none of the B‐cell antigens are specific for neoplastic LP cells; benign B‐cells also express them in the background. Among the many B‐cell antigens, OCT2 expression seen as a strong nuclear staining pattern in the LP cells is more robust than the rest of the B‐cell antigens. Among the GC markers, LP cells are negative for CD10 but consistently positive for BCL6. However, BCL6 is also positive in the TFH cells, often rosetting the LP cells. In our recent experience, we have found MEF2B expression being limited to neoplastic LP cells (except for remnant reactive GCs that can rarely be present). Other benign B‐ or T‐cells do not express MEF2B within the nodules of NLPHL. J‐chain expression is seen in the cytoplasm of the LP cells but can also be seen in plasma cells and rare other B‐cells. J‐chain staining of LP cells is less consistent than MEF2B, and being a cytoplasmic staining pattern, it does not highlight abnormal nuclear morphological features of the LP cells.

The MEF2B gene encodes for a protein that regulates the expression of the smooth muscle myosin heavy chain gene. MEF2B is a member of a family of transcription factors (MEF2A, MEF2B, MEF2C, and MEF2D) that are variably active in different tissues. MEF2B is strongly expressed in the GC B‐cells and modulates the expression of BCL6, another critical transcription factor that defines the GC B‐cell phenotype [6].

Two previous studies documented MEF2B expression in the LP cells of NLPHL [7, 8]. MEF2B expression was noted in 100% of the NLPHL cases, and Hodgkin Reed Sternberg cells of classic Hodgkin lymphoma were almost always negative for MEF2B expression. More recently, consistent expression of MEF2B has also been documented in Epstein Barr virus‐positive cases of NLPHL [9]. Despite these studies, the utility of MEF2B in the diagnostic algorithm of NLPHL is underappreciated. Here we describe an illustrative case with appropriate images.

A man in his thirties presented with a 6–12‐month history of right neck lymphadenopathy and no other symptoms. The excised lymph node showed a large nodular pattern and a polymorphous infiltrate composed predominantly of small lymphocytes and histiocytes and intermingled atypical large lymphoid cells with moderate amounts of cytoplasm, lobulated nuclei, and variably prominent nucleoli. Atypical cells had features of LP cells. These LP cells were positive for CD20, PAX5, BCL6, MEF2B, OCT2, and BOB1, and negative for CD3, CD15, MUM1, ALK‐1, and EBER (Figure 1). CD3 and PD‐1 highlighted frequent T‐cell rosettes surrounding the neoplastic cells. CD21 highlighted expanded follicular dendritic cell meshworks. A diagnosis of “Nodular lymphocyte predominant Hodgkin lymphoma, classical B‐cell‐rich nodular (Fan pattern A)” was rendered. MEF2B expression was noted in all the neoplastic LP cells, and the expression was restricted to the neoplastic cells. The abnormal nuclear morphology was also highlighted by the immunostain aiding the diagnosis. We have used MEF2B immunostain in evaluating cases of NLPHL during the last 2 years. We have appreciated strong MEF2B expression in the LP cells in 100% of the 12 recent cases tested, confirming previous observations.

**FIGURE 1:**
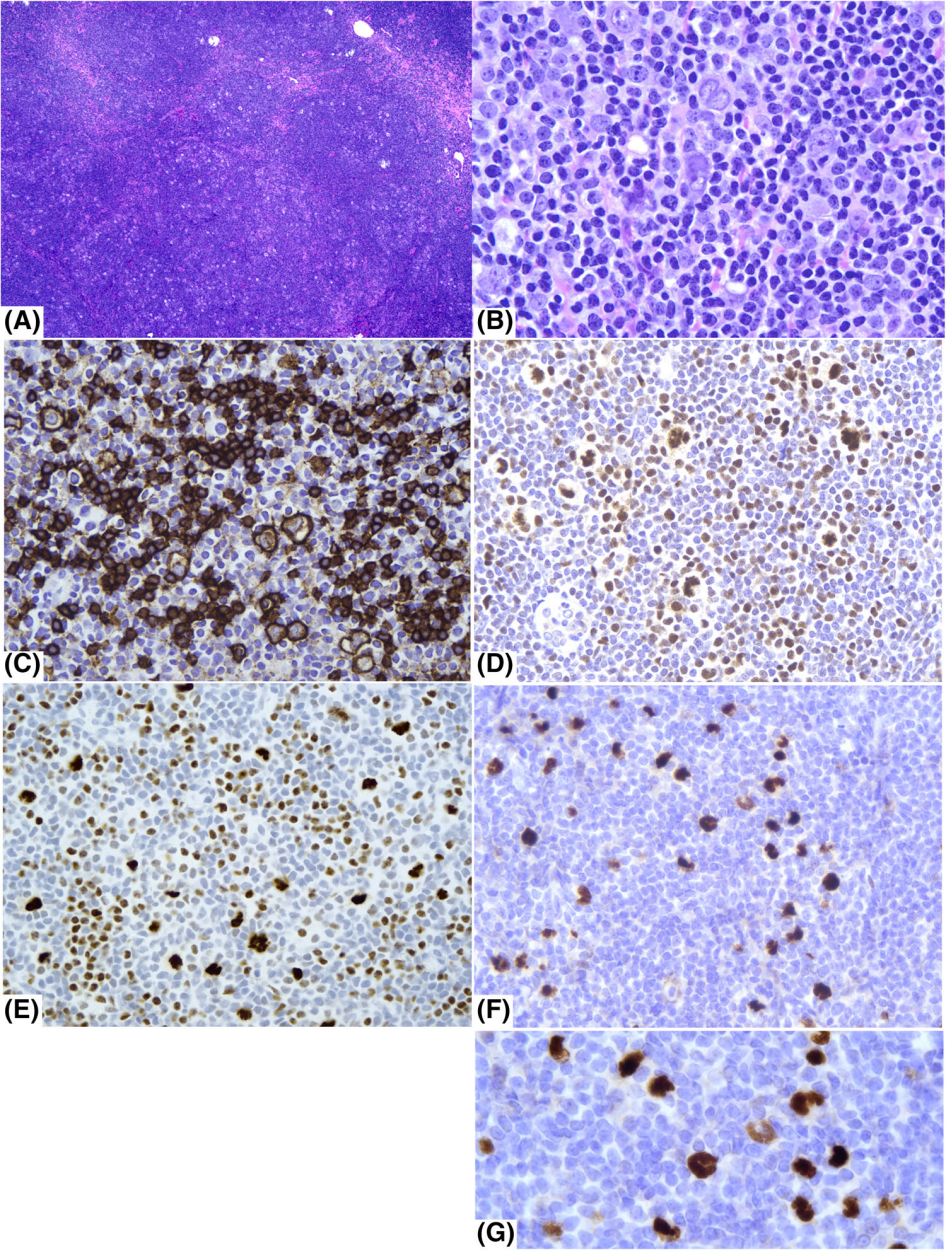
Lymph node shows a large nodular pattern and a polymorphous infiltrate of cells (A) (H&E X40); on higher magnification, small lymphoid cells, rare histiocytic cells, and scattered neoplastic LP cells with lobulated nuclei and small nucleoli are noted (B) (H&E X600). CD20 immunostain shows CD20 expression in the neoplastic LP cells and small benign B‐cells (C) (X400). BCK6 immunostain shows BCL6 expression in the neoplastic LP cells and small benign T‐cells, with some T‐cells rosetting the LP cells (D) (X400). OCT2 immunostain shows OCT2 expression in the neoplastic LP cells and small benign B‐cells (E) (X400). MEF2B immunostain shows MEF2B expression exclusively in the neoplastic LP cells (F; X400); higher magnification of the MEF2B stain highlights the abnormally lobulated nuclei of the LP cells.

Although we present the images from a case with a typical Fan pattern A (to convey the message more clearly), its utility is best appreciated among cases with Fan patterns C–F (seven of our cases had areas with Fan patterns C–F) [10]. We had one case with Fan pattern F across the entire lesion, where the MEF2B immunostain contributed immensely to arriving at the correct diagnosis. In the latter case, the neoplastic cells expressed CD30, and the expression of B‐cell antigens (CD20, CD79a, and PAX5) was weak and present only in a subset of cells; however, the neoplastic cells were uniformly positive for MEF2B along with expression of BOB1, OCT2, BCL6, MUM1, EMA (subset) without CD15, CD19, and EBER. The case also had many small B‐cells in the background contributing to the diagnostic challenge. Through this communication, we highlight the ease of recognizing neoplastic LP cells using MEF2B immunostain.

## AUTHOR CONTRIBUTIONS

Alireza Torabi: Evaluated the cases and critically reviewed the manuscript.

Jonathan R Fromm: Standardized the antibody and critically reviewed the manuscript.

Kikkeri N Naresh: Wrote the manuscript.

## CONFLICT OF INTEREST STATEMENT

None.

## FUNDING INFORMATION

None

## ETHICS STATEMENT

n/a.

## PATIENT CONSENT STATEMENT

n/a.

## Data Availability

Data sharing is not applicable to this article as no datasets were generated or analyzed during the current study.
